# Two-year home-based nocturnal noninvasive ventilation added to rehabilitation in chronic obstructive pulmonary disease patients: A randomized controlled trial

**DOI:** 10.1186/1465-9921-12-112

**Published:** 2011-08-23

**Authors:** Marieke L Duiverman, Johan B Wempe, Gerrie Bladder, Judith M Vonk, Jan G Zijlstra, Huib AM Kerstjens, Peter J Wijkstra

**Affiliations:** 1 Department of Pulmonary Diseases, University Medical Center Groningen, University of Groningen, Groningen, The Netherlands; 2 Department of Home Mechanical Ventilation, University Medical Center Groningen, University of Groningen, Groningen, The Netherlands; 3 Center for Rehabilitation, University Medical Center Groningen, University of Groningen, Groningen, The Netherlands; 4 Department of Epidemiology, University Medical Center Groningen, University of Groningen, Groningen, The Netherlands; 5 Department of Critical Care, University Medical Center Groningen, University of Groningen, Groningen, The Netherlands

## Abstract

**Background:**

The use of noninvasive intermittent positive pressure ventilation (NIPPV) in chronic obstructive pulmonary disease (COPD) patients with chronic hypercapnic respiratory failure remains controversial as long-term data are almost lacking.

The aim was to compare the outcome of 2-year home-based nocturnal NIPPV in addition to rehabilitation (NIPPV + PR) with rehabilitation alone (PR) in COPD patients with chronic hypercapnic respiratory failure.

**Methods:**

Sixty-six patients could be analyzed for the two-year home-based follow-up period. Differences in change between the NIPPV + PR and PR group were assessed by a linear mixed effects model with a random effect on the intercept, and adjustment for baseline values. The primary outcome was health-related quality of life (HRQoL); secondary outcomes were mood state, dyspnea, gas exchange, functional status, pulmonary function, and exacerbation frequency.

**Results:**

Although the addition of NIPPV did not significantly improve the Chronic Respiratory Questionnaire compared to rehabilitation alone (mean difference in change between groups -1.3 points (95% CI: -9.7 to 7.4)), the addition of NIPPV did improve HRQoL assessed with the Maugeri Respiratory Failure questionnaire (-13.4% (-22.7 to -4.2; p = 0.005)), mood state (Hospital Anxiety and Depression scale -4.0 points (-7.8 to 0.0; p = 0.05)), dyspnea (Medical Research Council -0.4 points (-0.8 to -0.0; p = 0.05)), daytime arterial blood gases (PaCO_2 _-0.4 kPa (-0.8 to -0.2; p = 0.01); PaO_2 _0.8 kPa (0.0 to 1.5; p = 0.03)), 6-minute walking distance (77.3 m (46.4 to 108.0; p < 0.001)), Groningen Activity and Restriction scale (-3.8 points (-7.4 to -0.4; p = 0.03)), and forced expiratory volume in 1 second (115 ml (19 to 211; p = 0.019)). Exacerbation frequency was not changed.

**Conclusions:**

The addition of NIPPV to pulmonary rehabilitation for 2 years in severe COPD patients with chronic hypercapnic respiratory failure improves HRQoL, mood, dyspnea, gas exchange, exercise tolerance and lung function decline. The benefits increase further with time.

**Trial registration:**

ClinicalTrials.Gov (ID NCT00135538).

## Background

Chronic obstructive pulmonary disease (COPD) is a progressive disease leading to severe dyspnea at low exercise levels, reduced health-related quality of life (HRQoL) and high mortality rates [1].

Pulmonary rehabilitation (PR) improves dyspnea, exercise capacity, and HRQoL in patients with COPD [2]. These positive effects can be maintained well if the exercise training is continued at home after initial intensive PR [3]. However, in severe COPD patients, PR may be difficult to perform, and effects may be less maintainable at home [4]. Therefore, there is a need for additive therapies enhancing the effectiveness of PR, especially in patients with severe COPD.

We recently showed that the addition of 3-month nocturnal noninvasive intermittent positive pressure ventilation (NIPPV) to an intensive multidisciplinary rehabilitation program improves the outcomes of PR in severe COPD patients with chronic hypercapnic respiratory failure [5]. Three other studies have also investigated noninvasive ventilation in combination with PR, but assessed short-term effects only [6-8]. A few studies showed conflicting results of long-term effects of NIPPV in COPD [9-11]. However, these studies did not add NIPPV to PR and ventilator settings used were probably too low to provide beneficial effects [12]. The present study explores whether the initial positive effects of 3-month NIPPV in addition to PR, with the use of sufficient ventilator settings, can be maintained over 2-year home-based follow-up in COPD patients with chronic hypercapnic respiratory failure. Outcome parameters were HRQoL, mood state, dyspnea scores, gas exchange, functional status, pulmonary function, and exacerbation frequency.

## Methods

### Patients

Patients with COPD GOLD stage III or IV [1] (forced expiratory volume in 1 second (FEV_1_)/forced vital capacity < 70% and FEV_1 _< 50% predicted), aged between 40 and 76 years, in stable clinical condition (no exacerbation in the four weeks prior to study participation together with a pH>7.35); and with chronic hypercapnic respiratory failure (an arterial carbon dioxide pressure (PaCO_2_) > 6.0 kPa at rest while breathing room air) were included. Exclusion criteria were: cardiac or neuromuscular diseases limiting exercise tolerance; previous exposure to a pulmonary rehabilitation program during the previous 18 months or previous exposure to chronic NIPPV ever; or an apnea/hypopnea index ≥ 10/hour. An overnight polygraphy (Embletta pds, Medcare Automation BV, Amsterdam, the Netherlands) was performed in all patients with a body mass index ≥ 30 kg/m^2^, and in patients who snored or had complaints of disrupted sleep, excessive daytime sleepiness, or morning headache. The study was approved by the local Medical Ethics Committee of the University Medical Centre Groningen, University of Groningen and was registered at ClinicalTrials.Gov (ID NCT00135538). All participants gave written informed consent to participate.

### Study design

#### Randomization

The study design was randomized controlled with parallel-groups. Patients were assigned to nocturnal NIPPV in addition to rehabilitation (NIPPV + PR) or to rehabilitation alone (PR). Randomization was computerized and performed by an independent statistician, with minimization for FEV_1 _(≤ 1.2 L or > 1.2 L), PaCO_2 _(≤ 7.0 kPa or > 7.0 kPa), and body mass index (≤ 30 kg/m^2 ^or > 30 kg/m^2^) [13].

#### Rehabilitation

After a 12-week multidisciplinary in-hospital rehabilitation program [5], all patients continued with a home-based rehabilitation program, with or without nocturnal NIPPV. In the current manuscript results of the home-based period are presented; results of the multidisciplinary in-hospital program have been reported separately [5]. The home-based program consisted of physiotherapy at a community practice 1-2 times a week during the whole study period, with or without home NIPPV. Most patients visited the physiotherapist two times a week. A few patients (both from the NIPPV + PR group and the PR group) visited the physiotherapist once a week because the distance to travel to the physiotherapy practice was too long. All participating physiotherapists in the study were members of the Northern COPD physiotherapists group, which means that the physiotherapists were regularly taught in COPD exercise programs, and work in a well-equipped environment for COPD patients.

Each session consisted of 30-minute periods of cycling exercises, walking, and inspiratory muscle training each. The cycling protocol consisted of intervals of one minute loaded cycling (aimed at 140% of a patient's initial peak work rate on cycle ergometry), and one minute unloaded cycling, during 30 minutes [14]. Inspiratory muscle training was performed on an inspiratory threshold device at an interval basis (two minutes of loaded breathing, followed by one minute rest), starting with the threshold resistance on 30% of baseline maximal inspiratory pressure (P_I_max), increasing the resistance with 5-10% per session until 70% P_I_max was reached [15]. In patients with low fat free mass, strength training was added. Patients were also instructed to stay as active as possible at home, they were stimulated to walk at least each day and to train with their inspiratory device. All sessions were noted in a diary in order to monitor the progress and attendances to the program. Furthermore, there was regular contact with the physiotherapists participating in this study. If patients did not show up without a good reason for a longer period they were regarded as drop-outs (3 patients in the PR group and 1 patient in the NIPPV + PR group). Oxygen was used during training to maintain arterial oxygen saturation >90%.

#### NIPPV

In the NIPPV + PR group, patients were instituted on nocturnal bilevel NIPPV. Noninvasive ventilation was supplied through a pressure cycled ventilator, applying both inspiratory and expiratory pressure (BiPAP; Synchrony, Respironics, INC., Murrysville, PA, USA). A nasal or full face mask (Mirage mask, ResMed Ltd, UK) of the proper size was used. The ventilator was set in a spontaneous/timed mode (S/T), with a backup frequency. Inspiratory positive airway pressure (IPAP) was increased up to maximal tolerated pressure and titrated towards an optimal correction of nocturnal arterial blood gases (PaCO_2_<6.0 kPa and arterial oxygen pressure (PaO_2_) >8.0 kPa). Effectiveness of NIPPV was initially monitored by means of arterial blood gas measurements during the night [5], during the home based period NIPPV effectiveness was monitored by means of transcutaneous O_2_-saturation and PCO_2_tc measurements performed with the TOSCA^® ^(Type TOSCA^® ^500, Linde Medical Sensors AG, Basel, Switzerland) [16,17]. Ventilator compliance was determined from the ventilator counter readings. A specialized nurse from our department of home mechanical ventilation supervised the home mechanical ventilation.

#### Outcomes

**Figure 1:**
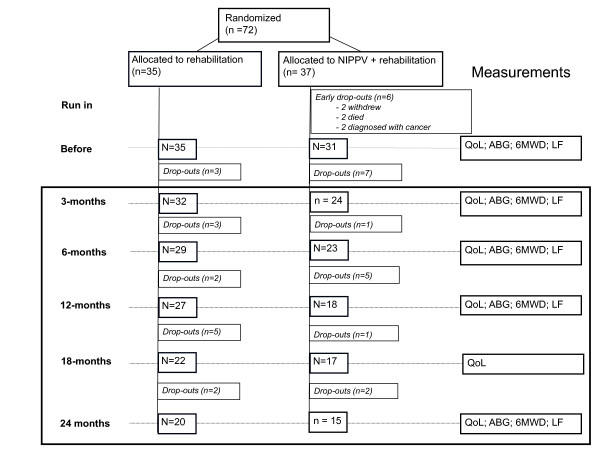
**Flow diagram of the study progress**. The present article presents the results of the home-based 3-24 month period, shown with a black square around it. QoL: health-related Quality of Life; ABG: arterial blood gases; 6MWD: 6-minute walking distance; LF: lung function measurements.

Outcome measures of the home-based period were performed just before the start of this period (after 3 months in-hospital rehabilitation), and then after 6, 12, 18, and after 24 months (Figure 1). The primary outcome was predefined to be HRQoL, assessed by the Chronic Respiratory Questionnaire (CRQ) [18]. Additionally, HRQoL was measured with the Maugeri Respiratory Failure questionnaire (MRF-28) [19], and Severe Respiratory Insufficiency questionnaire (SRI) [20]. Secondary outcomes were mood state (Hospital Anxiety and Depression scale (HADS) [21]), dyspnea scores (Medical Research Council (MRC) [22]), gas exchange (arterial blood gases), functional status (6-minute walking distance (6MWD), activity level (Groningen Activity and Restriction Scale (GARS) [23])), pulmonary function (FEV_1_, vital capacity, and lung volumes), and exacerbation frequency. An exacerbation was defined as an episode of increased pulmonary complaints for which (an increase in) oral steroids and/or antibiotics was needed (Figure 1). Details are given in the additional file 1.

### Sample size

To detect a clinically relevant change in the CRQ score of 10 points with 80% power, 40 patients per group were needed [24]. The target sample size was 50 patients per group, considering a probability of 20% drop-out of randomized patients.

### Analyses and Statistics

Continuous variables were summarized with the use of means and standard deviations or medians with interquartile ranges depending on their distribution. Treatment effects or differences in change between the PR and NIPPV + PR group, with the associated 95% CI and p-value, were assessed by a linear mixed effects model with a random effect on the intercept, with adjustment for the values at the start of the period [25]. Outcomes were screened for linearity by visual inspection of all plots. A full data set analysis was performed, signifying intention-to-treat, with all data of all patients available at the start of the home-based period included for analyses and all available data used for analyses until patients dropped out. A p < 0.05 was considered statistically significant. Analyses were performed by an independent statistician (JV) with SPSS 16.0.

## Results

### Patients

Thirty-two patients in the PR group and 24 patients in the NIPPV + PR group completed the 3-month multidisciplinary program [5], and were included in the present report (Figure 1, Table 1).

**Table 1:** Characteristics of the patients included at the start of the follow-up period.

Characteristics	NIPPV + rehabilitation	Rehabilitation
Subjects - n	24	32

Gender - M:F	16: 8	17: 15

Age - yrs, mean (SD)	63 (10)	61 (8)

Patients on LTOT - n (%)	14 (58%)	18 (56%)

BMI - kg/m^2^, mean (SD)	27.2 (5.1	27.0 (5.8

Active smokers, n (%)	5 (21%)	11 (34%)

Pack years - yrs, median (IQR)	42 (31-57)	43 (24-58)

Medication, n (%)		
inhaled corticosteroids	22 (92%)	29 (91%)
oral corticosteroids	10 (42%)	14 (44%)
bronch odilators	24 (100%)	31 (97%)
theophylline	5 (21%)	8 (25%)

**Figure 2:**
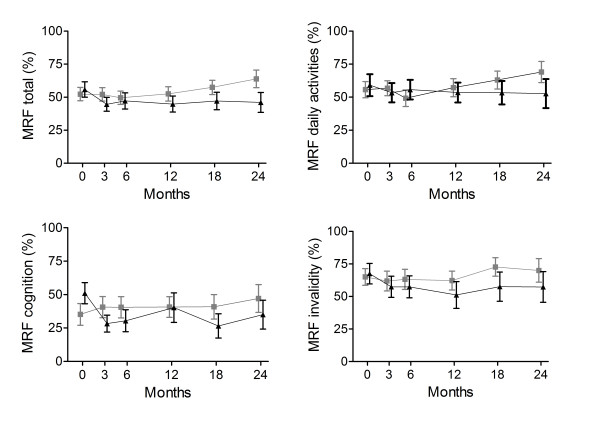
**Maugeri Respiratory Failure scores**. MRF scores at the different measurement points in the NIPPV + rehabilitation group (black triangles) and the rehabilitation group (grey blocks). Lower scores signify better quality of life. The change was significantly better in the NIPPV + rehabilitation group (p < 0.02).

**Figure 3:**
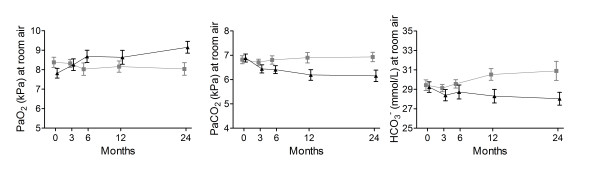
**Daytime arterial blood gases**. Daytime arterial blood gases without additional oxygen at the different measurement points in the NIPPV + rehabilitation group (black triangles) and the rehabilitation group (grey blocks). The change was significantly better in the NIPPV + rehabilitation group (p < 0.02).

**Figure 4:**
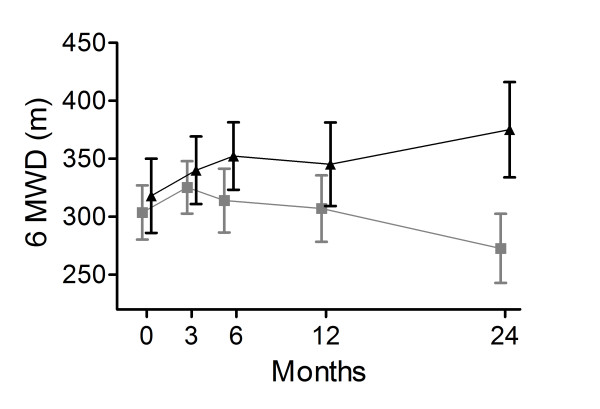
**6-minute walking distance**. 6MWD in meters at the different measurement points in the NIPPV + rehabilitation group (black triangles) and the rehabilitation group (grey blocks). The change was significantly better in the NIPPV + rehabilitation group (p < 0.001).

**Figure 5:**
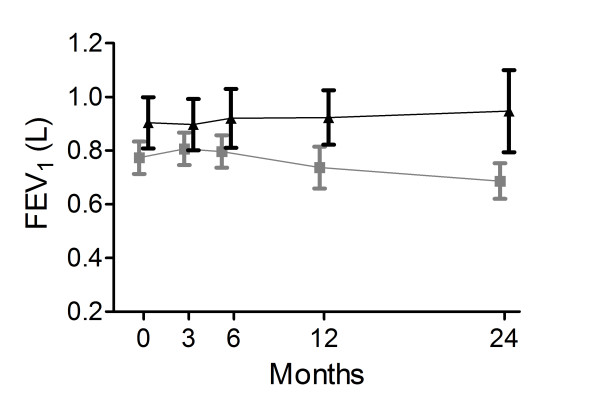
**Forced expiratory volume in 1 second (FEV_1_)**. FEV_1 _in liters (L) at the different measurement points in the NIPPV + rehabilitation group (black triangles) and the rehabilitation group (grey blocks). The change was significantly better in the NIPPV + rehabilitation group (p < 0.02).

Data are means (SD) or median (interquartile range, IQR), unless otherwise indicated. LTOT: long-term oxygen therapy; BMI: body mass index. Health-related quality of life scores, blood gases, exercise tolerance, and lung function data are presented in Figures 2-5 and additional file 1 tables 1-6.

Most patients suffered from one of more comorbidities, the most common being osteoporosis (NIPPV + PR group: 3 patients (13%); PR group: 4 patients (13%)); hypertension (NIPPV + PR group: 7 patients (29%); PR group: 8 patients (25%)); cardiac dysfunction and/or chronic atrial fibrillation (NIPPV + PR group: 8 patients (33%); PR group: 5 patients (16%)); depression (NIPPV + PR group: 4 patients (17%); PR group: 8 patients (25%)); and diabetes mellitus (PR group: 8 patients (25%)).

Diuretics were used by 6 patients in the NIPPV + PR group and 11 patients in the PR group at the start of the study period (not significantly different), but were started in significantly more patients in the PR group (NIPPV + PR group: 3 patients; PR group: 10 patients; p = 0.03), so that at the end of the study period significantly more patients in the PR group used diuretics compared to the NIPPV + PR group (p = 0.003).

At the start of the study period, 51 patients (91%) used inhaled corticosteroids, and 55 patients (98%) used bronchodilators (inhaled beta-agonist or anticholinergic medication) (Table 1). During the study period no further changes were made, except for the one patient in the PR group who initially did not want to use a bronchodilator but started on tiotropium during the follow up. At the start of the study period, 24 patients (43%) used oral corticosteroids (all at a standard dosage of 5 mg 3 times a week to 10 mg/day prescribed by their own pulmonologist to prevent exacerbations). Changes in oral steroid use were made in 6 patients: in 2 PR group patients oral steroids were started, in 3 PR group patients the dosage was increased, and in 1 PR patient oral steroids could be stopped. Thirteen patients (23%) were on theophylline, in one patient in the PR group theophylline was started during the study period. At the start of the study period 2 patients were on prophylactic antibiotics, during the study period azithromycin or doxycycline was started in an additional 3 patients in the NIPPV + PR group and 7 patients in the PR group (not significantly different).

### Treatment compliance and drop-outs for the complete study period

During the home-based follow-up period, nine patients in the NIPPV + PR group did not complete the study (three patients withdrew from follow-up, one patient had an aorta dissection, and five patients (21%) died; two from a COPD exacerbation, two suddenly at home without further cause verification, and one patient without further information). In the NIPPV + PR group, drop-outs had a significantly lower baseline PaO_2 _compared to completers (PaO_2 _7.2 (0.8) kPa vs. 8.2 (1.0) kPa; p = 0.02).

During the home-based period, 12 patients in the PR group did not complete the study (three patients were non-compliant, one received a lung transplantation, one got an ischemic stroke, one patient's clinical condition deteriorated making further measurements impossible, one was treated with CPAP by his own pulmonologist, and five patients (16%) died, all from a COPD exacerbation). In the PR group, at baseline, drop-outs had a significantly higher RV/%TLC ratio (63 (7) vs. 57 (8); p = 0.04), a worse 6MWD (232 (98) m vs. 347 (99) m; p = 0.004), and worse HRQoL (CRQ total, 69 (11) vs. 86 (20) points; p = 0.005) than those who completed the study.

There were no significant differences between the groups at the start of the study period (Table 1, additional file 1, Table S1 and Table S2), except for slightly better HRQoL scores in the NIPPV + PR group compared to the PR group (CRQ total score 96.8 (15.3) vs. 87.1 (18.9) points; p = 0.044; CRQ fatigue score 18.8 (3.9) vs. 15.4 (5.6) points, p = 0.015; SRI attendant symptoms: 71.1 (19.6) vs. 60.2 (19.6)%, p = 0.032. When the analysis was repeated with only patients who completed the whole study, there were no baseline differences. The number of patients that died during the study was the same in both groups (five patients).

### NIPPV settings

The mean IPAP at the start of the home-based follow-up period was 23 (4) cm H_2_O, with a mean EPAP of 6 (2) cm H_2_O, mean respiratory rate on NIPPV of 18 (3) breaths/min, an inspiration time of 1.0 (0.1) seconds, and a rise time of 1.2 (0.6) seconds. Fourteen patients used oxygen during the day (median flow rate of 2 L/min (range 0.75 to 4)), they also used oxygen while on the ventilator (median flow rate of 1.75 L/min (range 1 to 4 L/min)). Only minor adjustments were made during the study period in order to improve (daytime) arterial blood gases more. In 6 patients IPAP was increased by a median of 4 cm H_2_O (range 2 to 5 cm H_2_O), in three patients IPAP was decreased by a median of 2 cm H_2_O (range 1 to 3 cm H_2_O) to optimize comfort). Daytime of the nocturnal transcutaneous measurements (TOSCA^®^) are presented in additional file 1, Table S3. After two years, mean IPAP in the 15 remaining patients was 23 (4) cm H_2_O, mean EPAP 6 (2) cm H_2_O, mean respiratory rate on NIPPV 18 (3) breaths/min, inspiration time 0.9 (0.2) seconds, and rise time 1.2 (0.6) seconds. Seven patients used oxygen during the day (median flow rate of 1.5 L/min (range 1 to 3)), however only four of them needed oxygen when on the ventilator (median flow rate of 2 L/min (range 2 to 4 L/min)).

One patient was ventilated through a nose mask, the remaining through a full face mask. Compliance was good, after two years patients used their ventilator 94% of the days (range 75 to 100%), with a median use per day of 6.9 hours (range 40 minutes to 11.4 hours/24 hours).

### Health-related quality of life, mood state, and dyspnea

The change in CRQ total and domain scores did not differ between both groups (Table 2, for absolute numbers see additional file 1, Table S1). The MRF-28 total score, and its domains daily activities and invalidity, improved more in the NIPPV + PR group than the PR group (difference in change for MRF-28 total score: -13.4% (95% CI -22.7 to -4.2; p = 0.005), Figure 2, additional file 1, Table S4). The SRI physical functioning domain improved more in the NIPPV + PR group than the PR group (difference 10.7% (95% CI 3.8 to 17.6; p = 0.003)), additional file 1, Table S2). The HADS and MRC scores improved more in the NIPPV + PR group than the PR group (Table 3, for absolute numbers see additional file 1, Table S5).

**Table 2:** Changes in Chronic Respiratory Questionnaire

	Change up to 24 months
CRQ total - points	

N+R - mean (95% CI)	-3.6 (-10.1 to 2.9)

R - mean (95% CI)	-2.3 (-7.8 to 3.2)

Adjusted difference in change - mean (95% CI)*	-1.3 (-9.7 to 7.4)

CRQ dyspnea - points	

N+R - mean (95% CI)	-1.5 (-4.0 to 0.8)

R - mean (95% CI)	0.0 (-2.1 to 2.1)

Adjusted difference in change - mean (95% CI)*	-1.7 (-4.8 to 1.5)

CRQ fatigue - points	

N+R - mean (95% CI)	-1.5 (-3.6 to 0.4)

R - mean (95% CI)	-1.5 (-2.9 to 0.2)

Adjusted difference in change - mean (95% CI)*	-0.2 (-2.7 to 2.3)

CRQ emotion - points	

N+R - mean (95% CI)	-1.1 (-3.6 to 1.3)

R - mean (95% CI)	-0.4 (-2.5 to 1.7)

Adjusted difference in change - mean (95% CI)*	-0.8 (-4.0 to 2.5)

CRQ mastery	

N+R - mean (95% CI)	-0.8 (-2.5 to 0.6)

R - mean (95% CI)	-0.7 (-2.1 to 0.4)

Adjusted difference in change - mean (95% CI)*	0.0 (-2.1 to 2.1)

Data presented are mean changes (95% confidence intervals). * The differences in change are the treatment effects or between groups differences in change (95% CI), with adjustment for the baseline values. A positive difference in change signifies more improvement over time with NIPPV + PR relative to PR alone.

The CRQ (chronic respiratory questionnaire) contains a total score (score range from best (140) to worst (20)), and 4 different domains: dyspnea domain (score range from best (35) to worst (5)), fatigue domain (score range from best (28) to worst (4)), emotion domain (score range from best (49) to worst (7)), mastery domain score range from best (35) to worst (5)). N+R: NIPPV + rehabilitation group; R: rehabilitation group.

**Table 3:** Changes in Groningen Activity and Restriction Scale (GARS), Hospital Anxiety and Depression scale (HADS), and Medical Research Council (MRC)

	Change up to 24 months
GARS, total - points	

N+R - mean (95% CI)	0.6 (-1.9 to 3.4)

R - mean (95% CI)	4.6 (2.3 to 6.9) ^†^

Adjusted difference in change - mean (95% CI)*	-3.8 (-7.4 to -0.4)‡

HADS, total - points	

N+R - mean (95% CI)	-0.2 (-3.4 to 2.7)

R - mean (95% CI)	3.6 (1.3 to 5.9) ^†^

Adjusted difference in change - mean (95% CI)*	-4.0 (-7.8 to 0.0)‡

MRC - points	

N+R - mean (95% CI)	0.2 (-0.2 to 0.4)

R - mean (95% CI)	0.6 (0.4 to 0.8) ^†^

Adjusted difference in change - mean; 95% CI*	-0.4 (-0.8 to -0.0)‡

Data presented are mean changes (95% confidence intervals). * The differences in change are the treatment effects or between groups differences in change (95% CI), with adjustment for the baseline values. A negative outcome indicates benefit for the NIPPV + rehabilitation group compared to the rehabilitation group.

GARS: Groningen Activity and Restriction Scale (score range from best (18) to worst (72)); HADS: Hospital Anxiety and Depression scale (score range from best (0) to worst (42)); MRC: Medical Research Council dyspnea scale (score range best (1) to worst (5)); N+R: NIPPV + rehabilitation group; R: rehabilitation group.

^†^: p < 0.05, significant difference in change over time within a group or ‡ p < 0.05: significant difference in change between groups.

### Daytime arterial blood gases

Arterial blood gases improved more in the NIPPV + PR group than the PR group (PaO_2 _0.8 kPa (95% CI 0.0 to 1.5; p = 0.032); PaCO_2 _-0.4 kPa (95% CI -0.8 to -0.2; p = 0.011); HCO_3_^- ^- 2.7 mmol/L (95% CI -4.4 to -1.1; p = 0.002); Figure 3, additional file 1, Table S6).

### Functional status

The 6MWD was maintained in the NIPPV + PR group, while it deteriorated in the PR group, the difference in change being significant (77.3 m (95% CI 46.4 to 108.0; p < 0.001; Figure 4, additional file 1, Table S6).

The GARS scores improved more in the NIPPV + PR group than the PR group (Table 3, for absolute numbers see additional file 1, Table S5).

### Pulmonary function

In the NIPPV + PR group, mean FEV_1 _stabilized or even slightly increased from 0.89 to 0.95 over time, which was significantly different from the mean reduction in FEV_1 _from 0.81 to 0.69 L in the PR group, the difference between the groups being 115 ml (95% CI 19 to 211; p = 0.019; Figure 5, Table 4, for absolute numbers see additional file 1, Table S7). There was no difference in VC or RV/%TLC, although the latter was measured only until the 12-month time point. There was no difference in change in maximal inspiratory muscle pressure (P_I_max) between the groups (Table 4, additional file 1, Table S7).

**Table 4:** Changes in Pulmonary function

	Change up to 24 months
FEV_1 _- liters	

N+R - mean (95% CI)	-0.03 (-0.10 to 0.05)

R - mean (95% CI)	-0.14 (-0.20 to -0.08) ^†^

Adjusted difference in change - mean (95% CI)*	0.12 (0.02 to 0.21) ‡

VC - liters	

N+R - mean (95% CI)	-0.01 (-0.19 to 0.17)

R - mean (95% CI)	-0.20 (-0.35 to -0.04) ^†^

Adjusted difference in change - mean (95% CI)*	0.19 (-0.05 to 0.42)

RV/%TLC	

N+R - mean (95% CI)	0.8 (-5.3 to 7.1)

R - mean (95% CI)	0.8 (-4.4 to 6.1)

Adjusted difference in change - mean (95% CI)*	0.2 (-8.0 to 8.4)

P_I_max - kPa	

N+R - mean (95% CI)	1.1 (0.4 to 2.5)

R - mean (95% CI)	-0.6 (-1.9 to 0.6)

Adjusted difference in change - mean (95% CI)*	1.7 (-0.0 to 3.6)

Data presented are mean changes (95% confidence intervals). * The differences in change are the treatment effects or between groups differences in change (95% CI), with adjustment for the baseline values. Lung volumes and P_I_max were measured until 12 months only.

FEV_1_: forced expiratory volume in 1 second in L; VC: maximal vital capacity, L; RV%TLC: residual volume as a percentage of total lung capacity; P_I_max: maximal inspiratory pressure in kPa. N+R: NIPPV + rehabilitation group; R: rehabilitation group.

^†^: p < 0.05, significant difference in change over time within a group or ‡ p < 0.05: significant difference in change between groups.

### Exacerbation frequency

The median exacerbation frequency was 3.0 exacerbations/year in both groups, the median hospitalization rate varied between 0-2 hospitalizations/year; both were not significantly different over time or between groups. Also, the median number of hospitalization days/year was also not significantly different over time or between groups.

## Discussion

Our study shows for the first time that home-based NIPPV + PR provides long-term benefit as to HRQoL, mood state, dyspnea, gas exchange, exercise tolerance, and FEV_1 _over PR alone in patients with severe COPD with chronic hypercapnic respiratory failure.

We believe the present RCT to be unique being the first to show that the addition of NIPPV improves FEV_1 _over 2-year follow-up compared to rehabilitation alone. The rehabilitation group had an average decline in postbronchodilator FEV_1 _of 83 ml/yr, while in the NIPPV + PR group this was 17 ml/yr. Except for smoking cessation [26] and, in some studies, the use of inhaled corticosteroids [27], no interventions have been shown to slow down FEV_1 _decline in COPD. Notably, effects found with smoking cessation and inhaled corticosteroids were smaller compared to the difference found in our study of 66 ml/year, which is a large effect in these severe COPD patients. We speculate that NIPPV stabilizes FEV_1 _either by volume expansion and/or a decrease in airflow obstruction. We were unable to show volume expansion, as we did not show significant changes in vital capacity, lung volumes or hyperinflation. However, lung volumes were measured until 12 months, so that volume expansion could still have occurred during the last year. Independently from changes in lung volumes, FEV_1 _stabilization is probably caused by a decrease in airflow obstruction. We speculate that a reduction in hypercapnia achieved with NIPPV reduces salt and water retention thereby reducing air wall edema [28]. Although speculative, reduced air wall edema might also exhibit a positive effect on airway wall remodeling by reducing inflammation when muscle fibers become less overstretched. An increase of FEV_1 _at short-term has been previously reported in studies using high inflation pressures with significant reductions in hypercapnia [29,30]. The high pressures might be of essential importance to improve of lung function [12].

This is the first randomized clinical trial to demonstrate that NIPPV is effective in improving daytime arterial blood gases at the longer term. This requires that effective ventilation during the night was achieved. Although it is obvious that effective ventilation is the first condition that should be met with NIPPV, it appears that in most NIPPV studies rather low positive pressure were used, so that outcomes have often been difficult to interpret [6,8-11]. We believe that close monitoring during the night is essential in improving gas exchange and that higher pressures are important to achieve good compliance [29] and effective ventilation [12,29-31]. High compliance as we achieved is essential. This all will have contributed to the positive clinical effects we found.

Exercise tolerance remained stable in the NIPPV + PR group, while it deteriorated in the PR group. A gradual loss of exercise tolerance at long term has been shown before in moderate to severe COPD patients, despite a out-of-hospital maintenance rehabilitation program [3,32-34]. Probably, positive effects of NIPPV on arterial blood gases give patients a more favorable condition to train and thus prevent deterioration in their physical condition, thus stressing the importance of additional therapies in COPD patients with chronic respiratory failure at long term.

Although these outcomes are promising, we have to notify that the results of our primary outcome, HRQoL, showed uncertain results, with the primary endpoint, CRQ, not showing any improvement. However, in hindsight, we have debated whether the CRQ is the optimal instrument to assess HRQoL in patients with chronic respiratory failure. By contrast, the MRF-28 and SRI were especially developed for patients with chronic respiratory failure improved, and are therefore probably more responsive in these patients [12,35]. Furthermore, we showed improvements in dyspnea scores and depression scores, both being an important determinant of HRQoL.

Chronic long-term NIPPV is a costly intervention. In a next study it would be interesting to add a true costs-benefit-analysis, as this may play a role in the further implication of NIPPV in chronic COPD patients. We did not find a difference between groups in overall exacerbation frequency, hospitalization rate for a COPD exacerbation or the number of hospitalization days. However in our cohort exacerbations did not occur frequently and the majority of the exacerbations occurred in a minority of the patients, so that large inter-individual differences occurred and data were not normally distributed.

The present study has some limitations. We did not use sham-ventilation in our control group, hence patients and investigators were not blinded. Sham-ventilation is difficult to implement at home during the long study period. Secondly, only 72 patients were included while according to the power calculation 40 patients per group were needed to find a 10-point change in CRQ total score. Due to the difficult recruitment and financial constraints we were unable to further extend the inclusion period. This may have influenced our results due to a type-II error for false negative outcomes, such as might have occurred with the CRQ. This does not, however, affect the observed significant improvements in our study. Finally, our study was not powered to find a difference in survival. While survival benefit of noninvasive ventilation has been shown one controlled study [11], clear evidence of improved survival is still lacking and should be investigated in larger studies.

## Conclusions

In conclusion, the present study is the first RCT to show that, with long-term, 2-year NIPPV in addition to PR as compared to PR alone, positive effects can be maintained in HRQoL and gas exchange, while additional effects can be achieved in functional status (exercise tolerance), mood state, dyspnea scores, and FEV_1 _in severe COPD patients with chronic hypercapnic respiratory failure. Although larger long-term studies have to confirm our results and give additional evidence on survival benefit and cost-effectiveness, with the present study evidence is provided for a rational use of NIPPV as an additional intervention next to pulmonary rehabilitation in severe COPD patients with chronic hypercapnic respiratory failure. Close monitoring of ventilatory support and the use of sufficiently high inspiratory pressures are probably crucial in obtaining these positive effects. This study shows that interventions that need a long period to reach their maximal effect like NIPPV should be studied over a long time scale, especially in slowly progressive diseases like COPD. Beneficial effects may require much time to develop fully and can therefore easily be underestimated.

## List of abbreviations

AHI: Apnea/Hypopnea Index; BMI: Body Mass Index; CI: Confidence Interval; COPD: Chronic Obstructive Pulmonary Disease; CPAP: Continuous Positive Airway Pressure; CRQ: Chronic Respiratory Questionnaire; EPAP: Expiratory Positive Airway Pressure; FEV_1_: Forced Expiratory Volume in 1 second; GARS: Groningen Activity and Restriction Scale; GOLD: Global Initiative of Lung Disease; HCO_3_^-^: bicarbonate; HRQoL: Health Related Quality of Life; IPAP: Inspiratory Positive Airway Pressure; kPa: kilo pascal; MRC: Medical Research Council; MRF-28: Maugeri Respiratory Failure questionnaire; 6MWD: 6-minute walking distance; NIPPV: Noninvasive Intermittent Positive Pressure Ventilation; PaO_2_: partial arterial oxygen pressure; PaCO_2_: partial arterial carbon dioxide pressure; P_I_max: maximal inspiratory pressure; PR: Pulmonary Rehabilitation; RCT: Randomized Controlled Trial; RV: Residual Volume; SRI: Severe Respiratory Insufficiency questionnaire; TLC: Total Lung Capacity; VC: Vital Capacity.

## Competing interests

Dr. Duiverman, Dr. Wempe, Ms. Bladder, Dr. Zijlstra, and Dr. Kerstjens have no competing interests. Dr. Wijkstra has received research grants from Respironics in 2009, 2010, and 2011.

## Individual contributions of all authors

MD and GB were the principal investigators of the study. JW contributed in the design and conduction of the rehabilitation program. JV performed the statistical analyses. JZ participated in the setting of the NIPPV. HK and PW designed the study and were head investigators. All mentioned investigators participated in the writing of the article and approved the final version.

## Supplementary Material

Additional file 1 **Entitled "Two-year home-based nocturnal noninvasive ventilation added to rehabilitation in chronic obstructive pulmonary disease patients: a randomized controlled trial: measurement information and supplemental tables"**, contains additional information about the measurements used, and additional information about the results, including absolute changes per group and results of the nocturnal transcutaneous CO_2 _and SaO_2 _measurements (TOSCA^®^). Click here for file
