# Supplementary material for: Two-year home-based nocturnal noninvasive ventilation added to rehabilitation in chronic obstructive pulmonary disease patients: A randomized controlled trial
Source: Respir Res. 2011 Aug 23;12(1):112. doi: 10.1186/1465-9921-12-112 (PMC3182911; doi:10.1186/1465-9921-12-112)
Supplement: Additional file 1 — Entitled "Two-year home-based nocturnal noninvasive ventilation added to rehabilitation in chronic obstructive pulmonary disease patients: a randomized controlled trial: measurement information and supplemental tables", contains additional information about the measurements used, and additional information about the results, including absolute changes per group and results of the nocturnal transcutaneous CO2 and SaO2 measurements (TOSCA®). [file 1465-9921-12-112-S1.DOC]

**Additional file 1**

Two-year home-based nocturnal noninvasive ventilation added to rehabilitation in chronic obstructive pulmonary disease patients: a randomized controlled trial: measurement information and supplemental tables.

Marieke L. Duiverman, Johan B. Wempe, Gerrie Bladder, Judith M.Vonk, Jan G. Zijlstra, Huib A.M. Kerstjens, Peter J. Wijkstra.

**Additional file 1: Methods**

##### Measurements

The following measurements were performed at baseline and after the 3-months intervention period.

Health-related quality of life

The primary outcome parameter was health-related quality of life, assessed by the interviewed version of the Chronic Respiratory Questionnaire (CRQ) [1]. In addition, health-related quality of life was measured with the Maugeri Respiratory Failure questionnaire (MRF-28) [2], and the Severe Respiratory Insufficiency questionnaire (SRI) [3].

We used the interviewed version of the Chronic Respiratory Questionnaire (CRQ). It contains 20 items divided into four dimensions: dyspnea, fatigue, emotion, and mastery. The CRQ total scores range from 20 to 140, with higher scores indicating better HRQoL. Physical function is assessed by asking the patients to quantify their dyspnea during 5 frequently performed activities in daily life. They are asked to choose 5 activities from a list of 25 activities or they can mention activities not on the list. The CRQ dyspnea domain scores range from 5 to 35. Physical function is also assessed by 4 items related to fatigue and energy level. The CRQ fatigue domain scores range from 4 to 27. Emotional function, including the emotion and mastery dimensions, includes questions about frustration, depression, anxiety, panic, and fear for dyspnea. The CRQ emotion domain scores range from 7 to 49, those of the mastery domain range from 4 to 28 [1].

The MRF-28 consists of 3 separate domains and a total score. The daily activities domain contains 11 items related to dyspnea during daily activities and impairments in daily activities. The cognition domain contains 4 items related to memory function, attention and concentration tasks. The invalidity domain contains 5 items on self-image, social functioning and relationships. Furthermore, the total score contains additional items related to fatigue, depression and problems with treatment, giving a total of 28 items. Scores are coded as 1 (patient agrees with the item) or 0 (patient does not agree). Scores are than recalculated as a percentage of items with which the patient agrees. MRF-28 scores range from 0-100, with higher scores indicating worse HRQoL [2].

The SRI contains 49 items divided in 7 subscales related to respiratory complaints (8 items), physical functioning (6 items), attendant symptoms and sleep (7 items), social relationships (6 items), anxiety (5 items), psychological well-being (9 items), social functioning (8 items), and a summary scale. Items are scores from 1 to 5, 35 items are then recoded, and the mean score is calculated to a percentage. SRI scores on each domain and the summary scale range from 0-100, with higher scores indicating better HRQoL [3].

Dyspnea

Dyspnea was assessed by the Medical Research Council [4]. The MRC is a 5-point scale (1: only dyspneic during heavy exercise; 5: too dyspneic to leave the house) containing items on various physical activities that precipitate dyspnea. Patients were instructed to read the descriptive statements and then select the statement which fitted best.

Mood state

The hospital anxiety and depression scale (HADS) was used to assess mood state. It contains 7 items assessing anxiety and 7 items assessing depression [5]. Scores per item range from 0 (no anxiety/ no depression) to 3 (maximal anxiety/ depression). The HADS total score ranges from 0 (best) to 42 (worst).

Activities of daily living

Daily activity level was assessed by using the Groningen Activity and Restriction Scale (GARS) [6]. This scale contains 18 items assessing the degree of independence in (instrumental) activities of daily living. Scores per item range from 1 (completely independent without any substantial effort) to 4 (completely dependent/impossible to perform alone). The GARS total score ranges from 18 (best) to 72 (worst).

Exercise tests

*6-minute walking distance*

A 6-minute walking test was performed indoors, along a 40-meter flat, straight corridor, with the turnaround point marked with a cone. All patients had performed a practice test during the run-in period. Patients used their usual walking aids and, if applicable, their usual ambulatory oxygen therapy during the test. The test assistant gave standardised encouragements every 30 seconds and told the patient after 2 and after 4 minutes that he/she was 2 and 4 minutes on his/her way [7].

Lung Function

All patients performed lung function testing post bronchodilatation with 400 microgram salbutamol. After 3 months and after 12 months both spirometry and lung volumes were tested. At the 6 month time point and at 24 months only spirometry was performed (Figure 1).

Vital capacity and forced expiratory volume in 1 second (FEV_1_) were obtained by spirometry according to ERS criteria [8]. Out of a least three technically correct measurements, the highest value of at least two reproducible values was used (with ≤150 ml difference between those two measurements). Lung volumes, total lung capacity, functional residual capacity and residual volume, were measured by body plethysmography [9]. Furthermore, maximal inspiratory pressure (P_I_max) was measured at residual volume after maximal expiration. The P_I_max manoeuvre was repeated at least five times with one minute rest between the measurements until 3 readings were obtained with less than 10% variance between the measurements. Pressures had to be maintained at least 1 second (Masterscreen PFT, Viasys, Houten, the Netherlands) [10, 11].

**References**

1. Guyatt GH, Berman LB, Townsend M, Pugsley SO, Chambers LW: **A measure of quality of life for clinical trials in chronic lung disease.** *Thorax* 1987, **42**:773–778.
2. Carone M, Bertolotti G, Anchisi F, Zotti AM, Donner CF, Jones PW: **Analysis of factors that characterize health impairment in patients with chronic respiratory failure.** *Eur Respir J* 1999, **13**:1293–1300.
3. [Windisch W](http://www.ncbi.nlm.nih.gov/pubmed?term="Windisch W"%5BAuthor%5D), [Freidel K](http://www.ncbi.nlm.nih.gov/pubmed?term="Freidel K"%5BAuthor%5D), [Schucher B](http://www.ncbi.nlm.nih.gov/pubmed?term="Schucher B"%5BAuthor%5D), Baumann H, Wiebel M, Matthys H, Petermann F: **The Severe Respiratory Insufficiency (SRI) Questionnaire: a specific measure of health-related quality of life in patients receiving home mechanical ventilation.** *J Epidemiol* 2003, **56**:752–759.
4. Task group on surveillance for respiratory hazards in the occupational setting. Brooks SM (Chairman). Surveillance for respiratory hazards. *ATS News* 1982; **8**: 12–16.
5. Zigmond AS, Snaith RP: **The hospital anxiety and depression scale.** Acta Psychiatr Scand 1983, **67**:361–370.
6. Kempen GI, Suurmeijer TP: **The development of a hierarchical polychomotomous ADL-IADL scale for noninstitutionalized elders.** *Gerontologist* 1990, **30**:497–502.
7. Guyatt GH, Sullivan MJ, Thompson PJ, Fallen EL, Pugsley SO, Taylor DW, Berman LB: **The 6-minute walk: a new measure of exercise capacity in patients with chronic heart failure.** *Can Med Assoc J* 1985, **132**:919–923.
8. Miller MR, Hankinson J, Brusasco V, Burgos F, Casaburi R, Coates A, Crapo R, Enright P, van der Grinten CP, Gustafsson P, Jensen R, Johnson DC, MacIntyre N, McKay R, Navajas D, Pedersen OF, Pellegrino R, Viegi G, Wanger J; ATS/ERS Task Force: **Standardisation of spirometry.** *Eur Respir J* **2005,** **26**:**319–3**38.
9. Wanger J, Clausen JL, Coates A, Pedersen OF, Brusasco V, Burgos F, Casaburi R, Crapo R, Enright P, van der Grinten CP, Gustafsson P, Hankinson J, Jensen R, Johnson D, Macintyre N, McKay R, Miller MR, Navajas D, Pellegrino R, Viegi G: **Standardisation of the measurement of lung volumes**. *Eur Respir J* 2005, **26**:511–522.
10. American Thoracic Society/European Respiratory Society: **ATS/ERS Statement on respiratory muscle testing.** *Am J Respir Crit Care Med* 2002, **166**:518–624.
11. Black LF, Hyatt RE: **Maximal respiratory pressures: normal values and relationship to age and sex.** *Am Rev Respir Dis* 1969; **99**:692-702.

**Additional file 1, Table S1: Absolute numbers and changes in Chronic Respiratory Questionnaire (CRQ) scores.**

|  | **Start** | **6 months** | **12 months** | **18 months** | **24 months** | **Change up to 24 months** |
| --- | --- | --- | --- | --- | --- | --- |
| CRQ total – points |  |  |  |  |  |  |
| N+R – mean (SD or 95% CI) | 96.8 (15.3) | 94.9 (20.3) | 93.5 (16.5) | 89.9 (17.3) | 93.7 (17.5) | -3.6 (-10.1 to 2.9) |
| R – mean (SD or 95% CI) | 87.1 (18.9) | 86.3 (18.4) | 87.7 (19.1) | 88.7 (21.5) | 88.7 (20.9) | -2.3 (-7.8 to 3.2) |
| Adjusted difference in change – mean (95% CI)* |  |  |  |  |  | -1.3 (-9.7 to 7.4) |
| CRQ dyspnea – points |  |  |  |  |  |  |
| N+R – mean (SD or 95% CI) | 19.4 (6.4) | 21.6 (6.2) | 19.2 (4.1) | 19.2 (4.8) | 18.9 (4.5) | -1.5 (-4.0 to 0.8) |
| R – mean (SD or 95% CI) | 19.5 (5.6) | 17.6 (4.3) | 17.6 (5.7) | 19.4 (6.5) | 18.7 (6.1) | 0.0 (-2.1 to 2.1) |
| Adjusted difference in change – mean (95% CI)* |  |  |  |  |  | -1.7 (-4.8 to 1.5) |
| CRQ fatigue – points |  |  |  |  |  |  |
| N+R – mean (SD or 95% CI) | 18.8 (3.9) | 17.4 (4.5) | 16.9 (3.8) | 16.9 (5.3) | 17.2 (5.0) | -1.5 (-3.6 to 0.4) |
| R – mean (SD or 95% CI) | 15.4 (5.6) | 16.1 (5.3) | 16.0 (4.6) | 16.2 (5.2) | 15.3 (5.3) | -1.5 (-2.9 to 0.2) |
| Adjusted difference in change – mean (95% CI)* |  |  |  |  |  | -0.2 (-2.7 to 2.3) |
| CRQ emotion – points |  |  |  |  |  |  |
| N+R – mean (SD or 95% CI) | 36.3 (6.4) | 34.1 (9.2) | 35.3 (7.5) | 33.0 (8.8) | 35.0 (6.7) | -1.1 (-3.6 to 1.3) |
| R – mean (SD or 95% CI) | 32.8 (8.4) | 32.5 (8.0) | 33.3 (7.1) | 32.2 (8.7) | 34.5 (7.4) | -0.4 (-2.5 to 1.7) |
| Adjusted difference in change – mean (95% CI)* |  |  |  |  |  | -0.8 (-4.0 to 2.5) |
| CRQ mastery – points |  |  |  |  |  |  |
| N+R – mean (SD or 95% CI) | 22.5 (3.6) | 21.8 (4.8) | 22.0 (4.9) | 21.7 (4.4) | 22.5 (4.7) | -3.6 (-10.1 to 2.9) |
| R – mean (SD or 95% CI) | 20.3 (4.6) | 20.2 (4.8) | 20.9 (4.9) | 20.9 (5.0) | 20.3 (5.2) | -2.3 (-7.8 to 3.2) |
| Adjusted difference in change – mean (95% CI)* |  |  |  |  |  | -1.3 (-9.7 to 7.4) |

***Legend:*** Data presented are means (standard deviation) and mean changes (95% confidence intervals). * The differences in change are the treatment effects or between groups differences in change (95% CI), with adjustment for the baseline value. A positive difference in change signifies more improvement over time with NIPPV + PR relative to PR alone.

The CRQ (chronic respiratory questionnaire) contains a total score (score range from best (140) to worst (20)), and 4 different domains: dyspnea domain (score range from best (35) to worst (5)), fatigue domain (score range from best (28) to worst (4)), emotion domain (score range from best (49) to worst (7)), mastery domain score range from best (35) to worst (5)). N+R: NIPPV + rehabilitation group; R: rehabilitation group.

**Additional file 1 Table S2: Absolute numbers and change in Severe Respiratory Insufficiency (SRI) questionnaire scores.**

|  | **Start** | **6 months** | **12 months** | **18 months** | **24 months** | **Change up to 24 months** |
| --- | --- | --- | --- | --- | --- | --- |
| SRI, SS – % |  |  |  |  |  |  |
| N+R – mean (SD or 95% CI) | 60.1 (11.4) | 59.5 (14.4) | 60.5 (10.9) | 56.8 (12.7) | 59.0 (14.1) | -3.4 (-7.1 to 0.4) |
| R – mean (SD or 95% CI) | 55.6 (14.7) | 55.6 (15.2) | 55.8 (13.4) | 54.4 (11.8) | 53.1 (14.0) | -6.3 (-9.2 to -3.2)^†^ |
| Adjusted difference in change – mean (95% CI)* |  |  |  |  |  | 2.9 (-1.9 to 7.8) |
| SRI, RC – % |  |  |  |  |  |  |
| N+R – mean (SD or 95% CI) | 58.7 (12.8) | 57.6 (14.4) | 57.5 (10.2) | 52.7 (11.7) | 55.0 (17.0) | -6.1 (-10.9 to -1.5)^†^ |
| R – mean (SD or 95% CI) | 52.1 (16.5) | 53.8 (16.6) | 53.1 (14.3) | 51.8 (13.5) | 51.9 (12.8) | -5.3 (-9.0 to -1.7)^†^ |
| Adjusted difference in change – mean (95% CI)* |  |  |  |  |  | -0.8 (-6.9 to 5.0) |
| SRI, PF – % |  |  |  |  |  |  |
| N+R – mean (SD or 95% CI) | 40.9 (21.5) | 44.4 (16.1) | 45.6 (16.5) | 44.0 (17.4) | 43.7 (18.4) | -0.8 (-6.3 to 4.4) |
| R – mean (SD or 95% CI) | 42.0 (17.8) | 41.4 (20.3) | 41.7 (20.4) | 38.3 (18.3) | 34.6 (18.5) | -11.6 (-16.0 to -6.9)^†^ |
| Adjusted difference in change – mean (95% CI)* |  |  |  |  |  | 10.7 (3.8 to 17.6)‡ |
| SRI, AS – % |  |  |  |  |  |  |
| N+R – mean (SD or 95% CI) | 71.1 (16.1) | 68.2 (15.4) | 68.3 (15.3) | 62.3 (16.5) | 67.4 (18.9) | -5.3 (-10.5 to 0.2) |
| R – mean (SD or 95% CI) | 60.2 (19.6) | 58.7 (16.5) | 60.8 (18.0) | 63.6 (13.5) | 64.3 (18.7) | 0.8 (-3.4 to 5.3) |
| Adjusted difference in change – mean (95% CI)* |  |  |  |  |  | -6.1 (-13.0 to 0.8) |
| SRI, SR – % |  |  |  |  |  |  |
| N+R – mean (SD or 95% CI) | 64.5 (12.8) | 64.3 (17.9) | 65.7 (14.0) | 64.6 (15.7) | 64.7 (15.0) | -2,5 (-7.8 to 2.7) |
| R – mean (SD or 95% CI) | 65.5 (14.1) | 64.5 (16.8) | 65.3 (12.9) | 63.3 (18.3) | 62.5 (13.9) | -3.8 (-8.0 to 0.6) |
| Adjusted difference in change – mean (95% CI)* |  |  |  |  |  | 1.1 (-5.7 to 8.0) |
| SRI, AX – % |  |  |  |  |  |  |
| N+R – mean (SD or 95% CI) | 63.3 (17.3) | 61.7 (20.2) | 62.5 (17.1) | 55.9 (20.1) | 59.3 (19.7) | -5.7 (-11.6 to 0.4) |
| R – mean (SD or 95% CI) | 56.9 (22.1) | 59.0 (19.2) | 57.5 (14.5) | 54.5 (11.3) | 51.5 (17.3) | -9.7 (-14.7 to -4.6)^†^ |
| Adjusted difference in change – mean (95% CI)* |  |  |  |  |  | 4.0 (-3.8 to 11.8) |
| SRI, WB – % |  |  |  |  |  |  |
| N+R – mean (SD or 95% CI) | 68.1 (13.9) | 65.0 (20.1) | 65.3 (11.9) | 63.9 (15.8) | 67.1 (17.6) | -1.1 (-6.7 to 4.4) |
| R – mean (SD or 95% CI) | 58.8 (19.1) | 60.4 (19.2) | 61.1 (14.6) | 58.2 (17.4) | 57.4 (18.7) | -5.5 (-9.9 to -1.1)^†^ |
| Adjusted difference in change – mean (95% CI)* |  |  |  |  |  | 4.4 (-2.9 to 11.6) |
| SRI, SF – % |  |  |  |  |  |  |
| N+R – mean (SD or 95% CI) | 54.1 (15.5) | 55.1 (19.5) | 58.4 (18.4) | 53.9 (16.5) | 55.6 (18.0) | -0.8 (-6.7 to 5.0) |
| R – mean (SD or 95% CI) | 53.6 (17.7) | 51.6 (20.6) | 50.9 (22.4) | 51.3 (19.3) | 49.2 (20.0) | -8.6 (-13.7 to -3.6)^†^ |
| Adjusted difference in change – mean (95% CI)* |  |  |  |  |  | 7.6 (-0.0 to 15.3) |

***Legend:*** Data presented are means (standard deviation) and changes (95% confidence intervals). * The differences in change are the treatment effects or between groups differences in change (95% CI), with adjustment for the baseline values. A positive difference in change number signifies more improvement over time with NIPPV + PR relative to PR alone.

^†^: p < 0.05, significant difference in change over time within a group or ‡ p < 0.05: significant difference in change between groups.

SRI: severe respiratory questionnaire, domains: respiratory complaints (RC), physical functioning (PF), attendant symptoms and sleep (AS), social relationships (SR), anxiety (AX), psychological well-being (WB), social functioning (SF) and a summary scale (SS); with all score ranges from best (100) to worst (0%).

N+R: NIPPV + rehabilitation group; R: rehabilitation group.

**Additional file 1 Table S3: Results of nocturnal transcutaneous measurements after 9 months out-of-hospital rehabilitation.**

|  | NIPPV + rehabilitation group | Rehabilitation group | p-value |
| --- | --- | --- | --- |
| PtcCO_2_, (kPa), mean (SD) | 6.91 (1.16) | 7.80 (1.17) | 0.04 |
| O_2_-saturation, mean (SD) | 94 (2) | 93 (4) | 0.60 |

***Legend:*** Shown are means (SD). PtcCO_2_: transcutenous carbon dioxide pressure

**Additional file 1 Table S4:** Changes in Maugeri Respiratory Failure (MRF-28) questionnaire scores.

|  | Change up to 24 months |
| --- | --- |
| MRF-28, total score – % |  |
| N+R – mean (95% CI) | 3.8 (-3.4 to 11.1) |
| R – mean (95% CI) | 17.2; (11.6 to 23.1)^†^ |
| Difference in change – mean (95% CI)* | -13.4 (-22.7 to -4.2)**‡** |
| MRF-28, daily activities – % |  |
| N+R – mean (95% CI) | 1.9 (-8.0 to 11.8) |
| R – mean (95% CI) | 20.2 (11.8 to 28.1)^†^ |
| Difference in change – mean (95% CI)* | -18.1 (-31.0 to -5.3)**‡** |
| MRF-28, cognition – % |  |
| N+R – mean (95% CI) | 4.2 (-8.2 to 16.4) |
| R – mean (95% CI) | 8.4 (-1.7 to 18.5) |
| Difference in change – mean (95% CI)* | -4.2 (-20.4 to 12.0) |
| MRF-28, invalidity – % |  |
| N+R – mean (95% CI) | 0.6 (-9.7 to 10.9) |
| R – mean (95% CI) | 16.2 (7.6 to 24.8)^†^ |
| Difference in change – mean (95% CI)* | -15.5 (-29.0 to -2.3)**‡** |

***Legend:*** Data presented are mean changes (95% confidence intervals). * The differences in change are the treatment effects or between groups differences in change (95% CI), with adjustment for the baseline values. A negative number signifies more improvement over time with NIPPV + PR relative to PR alone.

The Maugeri Respiratory failure (MRF-28) questionnaire contains a total score and 3 different domains; daily activities, cognition and invalidity, with all score ranges from best (0) to worst (100%).

^†^: p < 0.05, significant difference in change over time within a group or ‡ p < 0.05: significant difference in change between groups. For absolute numbers see Figure 2.

**Additional file 1 Table S5: Absolute numbers and changes in Groningen Activity and Restriction Scale (GARS), Hospital Anxiety and Depression scale (HADS), and Medical Research Council (MRC)**

|  | **Start** | **6 months** | **12 months** | **18 months** | **24 months** | **Change up to 24 months** |
| --- | --- | --- | --- | --- | --- | --- |
| GARS, total – points |  |  |  |  |  |  |
| N+R – mean (SD or 95% CI) | 39.1 (13.6) | 39.3 (11.6) | 38.2 (10.8) | 37.4 (10.8) | 36.5 (12.4) | 0.6 (-1.9 to 3.4) |
| R – mean (SD or 95% CI) | 40.0 (11.6) | 39.2 (11.9) | 40.5 (13.3) | 41.0 (12.5) | 38.5 (10.8) | 4.6 (2.3 to 6.9) ^†^ |
| Adjusted difference in change – mean (95% CI)* |  |  |  |  |  | -3.8 (-7.4 to -0.4)**‡** |
| HADS, total – points |  |  |  |  |  |  |
| N+R – mean (SD or 95% CI) | 10.8 (7.7) | 12.4 (9.5) | 9.9 (6.5) | 11.0 (7.7) | 11.3 (8.4) | -0.2 (-3.4 to 2.7) |
| R – mean (SD or 95% CI) | 15.3 (10.0) | 12.9 (7.7) | 13.4 (8.9) | 15.1 (7.3) | 16.5 (8.1) | 3.6 (1.3 to 5.9) ^†^ |
| Adjusted difference in change – mean (95% CI)* |  |  |  |  |  | -4.0 (-7.8 to 0.0)**‡** |
| MRC – points |  |  |  |  |  |  |
| N+R – mean (SD or 95% CI) | 2.9 (1.0) | 3.1 (1.0) | 3.0 (1.0) | 3.2 (0.9) | 2.9 (1.1) | 0.2 (-0.2 to 0.4) |
| R – mean (SD or 95% CI) | 3.2 (0.9) | 3.3 (1.1) | 3.4 (1.0) | 3.5(0.9) | 3.7 (1.0) | 0.6 (0.4 to 0.8) ^†^ |
| Adjusted difference in change – mean (95% CI)* |  |  |  |  |  | -0.4 (-0.8 to -0.0)**‡** |

***Legend:*** Data presented are means (standard deviation) and mean changes (95% confidence intervals). * The differences in change are the treatment effects or between groups differences in change (95% CI), with adjustment for the baseline values. A negative outcome indicates benefit for the NIPPV + rehabilitation group compared to the rehabilitation group.

GARS: Groningen Activity and Restriction Scale (score range from best (18) to worst (72)); HADS: Hospital Anxiety and Depression scale (score range from best (0) to worst (42)); MRC: Medical Research Council dyspnea scale (score range best (1) to worst (5)); N+R: NIPPV + rehabilitation group; R: rehabilitation group.

^†^: p < 0.05, significant difference in change over time within a group or **‡** p < 0.05: significant difference in change between groups.

Additional file 1 Table S6: Change in arterial blood gases and 6-minute walking distance

|  | **Change up to 24 months** |
| --- | --- |
| PaO_2_ – kPa |  |
| N+R – mean (95% CI) | 0.6 (0.0 to 1.1) ^†^ |
| R – mean (95% CI) | -0.2 (-0.6 to 0.2) |
| Difference in change – mean (95% CI)* | 0.8 (0.0 to 1.5)**‡** |
| PaCO_2_ – kPa |  |
| N+R – mean (95% CI) | -0.2 (-0.4 to 0.0) |
| R – mean (95% CI) | 0.2 (0.0 to 0.6) ^†^ |
| Difference in change – mean (95% CI)* | -0.4 (-0.8 to -0.2)**‡** |
| HCO_3_^-^ – mmol/L |  |
| N+R – mean (95% CI) | -0.4 (-1.7 to 0.8) |
| R – mean (95% CI) | 2.1 (1.1 to 3.4) ^†^ |
| Difference in change – mean (95% CI)* | -2.7 (-4.4 to -1.1)**‡** |
| 6MWD – meters |  |
| N+R – mean (95% CI) | -4 (-28 to 19) |
| R – mean (95% CI) | -82 (-103 to 62) ^†^ |
| Difference in change – mean (95% CI)* | 77 (46 to 108)**‡** |

***Legend:*** Data presented are mean changes; 95% confidence intervals. * The differences in change are the between groups differences in change (95% CI), with adjustment for the baseline values. A positive difference in change number signifies more improvement over time with NIPPV + PR relative to PR alone.

^†^: p < 0.05, significant difference in change over time within a group or **‡** p < 0.05: significant difference in change between groups. For absolute values see figure 3 and 5.

Blood gases were measured at daytime at rest without ventilation (for at least 4 hours) or additional oxygen (for at least 15 minutes). PaO_2_: partial oxygen pressure (kPa), PaCO_2_: partial carbon dioxide pressure; HCO_3_^-^: bicarbonate; 6MWD: 6-minute walking distance.

**Additional file 1 Table S7:** Absolute numbers and changes in pulmonary function.

|  | **Start** | **6 months** | **12 months** | **18 months** | **24 months** | **Change up to 24 months** |
| --- | --- | --- | --- | --- | --- | --- |
| FEV_1_ – litres |  |  |  |  |  |  |
| N+R – mean (SD or 95% CI) | 0.89 (0.39) | 0.92 (0.43) | 0.92 (0.35) | - | 0.95 (0.48) | -0.03 (-0.10 to 0.05) |
| R – mean (SD or 95% CI) | 0.81 (0.29) | 0.80 (0.27) | 0.74 (0.33) | - | 0.69 (0.25) | -0.14 (-0.20 to -0.08) ^†^ |
| Adjusted difference in change – mean (95% CI)* |  |  |  |  |  | 0.12 (0.02 to 0.21)**‡** |
| VC – litres |  |  |  |  |  |  |
| N+R – mean (SD or 95% CI) | 2.98 (0.89) | 2.88 (0.78) | 2.86 (0.84) | - | 2.98 (0.85) | -0.01 (-0.19 to 0.17) |
| R – mean (SD or 95% CI) | 2.62 (0.86) | 2.60 (0.66) | 2.57 (0.81) | - | 2.58 (0.73) | -0.20 (-0.35 to -0.04) ^†^ |
| Adjusted difference in change – mean (95% CI)* |  |  |  |  |  | 0.19 (-0.05 to 0.42) |
| RV/%TLC |  |  |  |  |  |  |
| N+R – mean (SD or 95% CI) | 55 (10) | - | 54 (9) | - | - | 0.8 (-5.3 to 7.1) |
| R – mean (SD or 95% CI) | 58 (9) | - | 58 (10) | - | - | 0.8 (-4.4 to 6.1) |
| Adjusted difference in change – mean (95% CI)* |  |  |  |  |  | 0.2 (-8.0 to 8.4) |
| P_I_max – kPa |  |  |  |  |  |  |
| N+R – mean (SD or 95% CI) | 6.44 (2.25) | 6.68 (2.44) | 6.97 (2.32) | - | - | 1.1 (0.4 to 2.5) |
| R – mean (SD or 95% CI) | 5.90 (2.31) | 6.12 (1.57) | 5.68 (1.99) | - | - | -0.6 (-1.9 to 0.6) |
| Adjusted difference in change – mean (95% CI)* |  |  |  |  |  | 1.7 (-0.0 to 3.6) |

***Legend:*** Data presented are means (standard deviation) and changes (95% confidence intervals). * The differences in change are the treatment effects or between groups differences in change (95% CI), with adjustment for the baseline values.

FEV_1_: forced expiratory volume in 1 second in L; VC: maximal vital capacity, L; RV%TLC: residual volume as a percentage of total lung capacity; P_I_max: maximal inspiratory pressure in kPa. N+R: NIPPV + rehabilitation group; R: rehabilitation group.

^†^: p < 0.05, significant difference in change over time within a group or **‡** p < 0.05: significant difference in change between groups.
